# Cervical Ripening in The Netherlands: A Survey

**DOI:** 10.1155/2013/745159

**Published:** 2013-06-26

**Authors:** Claartje M. A. Huisman, Marta Jozwiak, Jan Willem de Leeuw, Ben Willem Mol, Kitty W. M. Bloemenkamp

**Affiliations:** ^1^Department of Obstetrics and Gynecology, MC Haaglanden, 2512 VA The Hague, The Netherlands; ^2^Department of Obstetrics, K6-P-35, Leiden University Medical Center, P.O. Box 9600, 2300 RC Leiden, The Netherlands; ^3^Department of Obstetrics and Gynecology, Ikazia Hospital, 3008 AA Rotterdam, The Netherlands; ^4^Department of Obstetrics and Gynecology, Amsterdam Medical Center, 1105 AZ Amsterdam, The Netherlands

## Abstract

*Objective*. We aim to investigate methods and use of cervical ripening in women without and with a prior cesarean delivery in The Netherlands. *Methods*. In 2010, we conducted a postal survey in all Dutch hospitals with a labor ward. One gynecologist per hospital was addressed and was asked to respond on behalf of the staff. The questionnaire contained 31 questions concerning cervical ripening and induction of labor. We compared this survey to a similar Dutch survey conducted in 2006. *Results*. Response rate was 78% (70/92 hospitals). In women without a prior cesarean and in need of cervical ripening, all hospitals (100%) applied prostaglandins (either E1 or E2). In women with a prior cesarean, 21.4% of the hospitals performed an elective cesarean section if delivery was indicated (26.0% in 2006). In case of cervical ripening, 72.7% used mechanical methods (49.1% in 2006), 20.0% used prostaglandins (40.4% in 2006), 3.6% used a combination of prostaglandins and mechanical methods, and 3.6% used membrane-sweeping or oxytocin. *Conclusions*. In 2010, in The Netherlands, prostaglandins and Foley catheters were the preferred methods for cervical ripening in women without and with a prior cesarean, respectively. Use of mechanical methods in women with a prior cesarean has increased rapidly between 2006 and 2010, corresponding with decreasing use of prostaglandins and elective repeat cesarean sections.

## 1. Introduction

In 2007, 33% of all deliveries in The Netherlands were induced [1]. It is estimated that more than half of the women in whom labor is induced have an unfavorable cervix, defined as a Bishop score less than 6 [2]. The national guidelines of the Dutch Society of Obstetrics and Gynecology (NVOG), the American College of Obstetricians and Gynecologists (ACOG), the Royal College of Obstetricians and Gynaecologists (RCOG), and the Society of Obstetricians and Gynaecologists of Canada (SOGC) advise the use of prostaglandins for cervical ripening in this group of women [3–6]. As an alternative, these guidelines mention that a Foley catheter can be used. However, the RCOG guideline states that mechanical methods should not be used routinely, and the SOGC guideline conveys that more data are needed to be able to draw firm conclusions about their effectiveness [5, 6].

20% of all women with a prior cesarean delivery that attempt a trial of labor in the subsequent pregnancy are induced [7]. According to the Dutch (NVOG) guideline on induction of labor, caution is advocated concerning the use of contraction-stimulating drugs in women with a prior cesarean section [8]. Internationally, there seems to be a lack of consensus on the use of prostaglandins in women with a prior cesarean delivery. The 2007 RCOG guideline recommends a limit of 6 mg for prostaglandin cervical ripening [5]. The 2004 ACOG guideline states that appropriate case selection and avoiding sequential prostaglandin and oxytocin use offers the lowest risk of uterine rupture [9, 10]. The 2005 SOGC guideline advocates that prostaglandins should only be used in exceptional situations and after appropriate counselling [6]. Mechanical methods for cervical ripening such as the Foley catheter seem to be a good alternative in women with a prior cesarean delivery, carrying a lower risk of uterine rupture compared to prostaglandins [11–13].

In The Netherlands, both pharmacological and mechanical methods of cervical ripening are applied, both in women with and without a prior caeearean delivery. However, results from the (2006) survey by Reijers et al. showed that mechanical methods were rarely used [14]. 

Due to the (re)introduction of the Foley catheter in a randomised controlled trial, the PROBAAT trial, conducted in the Netherlands between February 2009 and May 2010, awareness about and use of the Foley catheter have presumably increased, especially in women with a prior cesarean delivery. The PROBAAT trial comparing Foley catheter and prostaglandins for cervical ripening in women without a prior cesarean delivery showed that both methods are equally effective with less maternal and neonatal side effects when using the Foley catheter [15]. 

We conducted a nationwide survey to assess current practice of cervical ripening in women with and without a prior cesarean delivery in 2010. Results of the PROBAAT trial were unknown at time of the survey. 

## 2. Material and Methods

In April 2010, all 92 Dutch hospitals with an obstetric practice received a postal questionnaire concerning methods of cervical ripening in women with an unfavorable cervix. One obstetrician per hospital was addressed and was asked to reply on behalf of the hospitals obstetric staff and based on protocols or policies. The survey consisted of two sections concerning women without (part one) and with (part two) a history of a cesarean delivery (S1). A total of 31 multiple choice questions with the opportunity for additional comments were given. Both sections contained questions concerning preferred method of cervical ripening, frequency, and maximum daily dose of medication. Additionally, we inquired if a difference in treatment was made between nulli- and multiparous women. Respondents were asked whether a cervical scoring system, such as the Bishop score, was used in decisions concerning labor induction and cervical ripening [2]. Methods of maternal and fetal monitoring and subsequent treatment after one and two days of cervical ripening were inquired. In part two, respondents were additionally asked if there were any reasons not to induce labor or apply cervical ripening in women with a prior cesarean delivery. 

Nonrespondents received a reminder email after six weeks and, if necessary, a phone call two weeks later.

Our results were compared to the (2006) survey on induction of labor in women with an unfavorable cervix by Reijers et al. for both women without and with a prior cesarean delivery [14]. The questions posed in the 2010 survey were mostly consistent with those in the 2006 survey.

 We compared the use of cervical ripening and methods for cervical ripening between 2006 and 2010. For categorical or dichotomous data differences were tested using the chi-square test. All analyses were performed using SPSS version 17.0 (SPSS Inc, Chicago, IL, USA).

## 3. Results

Of the 92 surveys sent 70 were returned, giving a response rate of 78%. Respondents according to type of hospital are shown in Table 1.

### 3.1. Part One: Women without a Prior Cesarean Delivery

#### 3.1.1. Cervical Ripening Methods

 An overview of the various methods of cervical ripening of the unfavorable cervix in women without a prior cesarean delivery is shown in Table 2. Also, doses and maximum frequency of administration are specified for the preferred method of cervical ripening. Twenty-five hospitals (36%) used more than one method for cervical ripening. If applicable, the second preferred method is also shown. Two hospitals adjusted dose of vaginal prostaglandin E2 gel according to parity.

**Figure 1:**
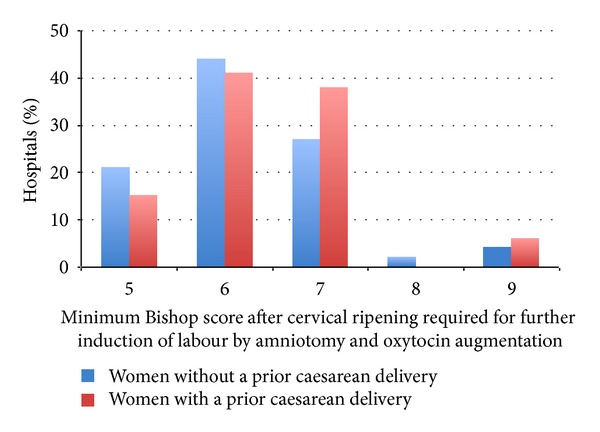
Minimum Bishop score after cervical ripening required for induction of labor by amniotomy and oxytocin augmentation in women without and with a prior cesarean delivery in The Netherlands in 2010. Missing value: *n* = 1.

For the assessment of cervical ripeness and the consequent decision of cervical ripening and induction, 48 hospitals (69%) used a cervical scoring system such as the Bishop score. The remaining hospitals assessed cervical ripeness using a vaginal examination, without a specified scoring system. Of the 47 hospitals that used the Bishop score, induction after cervical ripening by amniotomy and oxytocin augmentation was performed at a certain Bishop score (Figure 1). Six of the 22 hospitals (27%) that did not use the Bishop score performed amniotomy at a lower “score” of their own scoring system in multiparous compared to nulliparous women when a “favorable” cervix was found by vaginal examination. 

All hospitals applied fetal monitoring at set times after starting cervical ripening. Two hospitals (3%) applied continuous fetal monitoring with cardiotocography (CTG) during cervical ripening with vaginal prostaglandin E2 gel (1 and 2 mg). Induction of labor by amniotomy and subsequent oxytocin augmentation after proven cervical ripeness was performed at any moment of the day in 35 hospitals (50%), the morning after the start of cervical ripening in 29 hospitals (41%), and depended on the indication of induction in 5 hospitals (9%). 

The most reported reasons for the preferred method of cervical ripening were “ease in use” (57%), “reduced hyperstimulation risk” (24%), and “increased likelihood of delivery within 24 hours” (19%). The arguments “ease in use” and “reduced hyperstimulation risk” were predominantly mentioned by hospitals that used prostaglandin E2 gel or a slow release prostaglandin E2 vaginal insert. The argument “more deliveries in 24 hours” was given by all three hospitals that use the Foley catheter in combination with prostaglandins. Other important arguments were “tradition,” “experience” and, among slow release prostaglandin E2 vaginal insert users, “the possibility of removing it.”

The policy after insufficient result of cervical ripening after one or two days is shown in Table 3.

### 3.2. Part Two: Women with a Prior Cesarean Delivery

#### 3.2.1. Reasons for Not Inducing Labor in Women with a Prior Cesarean Delivery

None of the hospitals strived for a vaginal birth after cesarean (VBAC) in women with a classical incision at the prior cesarean delivery, and 53 hospitals (76%) did not pursue a VBAC in women with more than one prior cesarean delivery. A prior cesarean delivery before a gestational age of 34 weeks and no prior vaginal deliveries were relevant in the decision not to attempt a trial of labor in two hospitals.

When induction of labor was required, VBAC was not pursued in nonvertex position in 51 hospitals (73%), in an unfavorable cervix in 28 hospitals (40%), in a nonengaged head in 25 hospitals (36%), or in a twin pregnancy in 11 hospitals (16%). 

 In cases in which a prompt delivery was desired, eight hospitals (11%) performed an elective cesarean section. When labor was induced in women with a prior cesarean delivery, 14 hospitals (20%) had a different policy concerning induction of labor in women with and without a prior vaginal delivery. 

#### 3.2.2. Cervical Ripening Methods

Cervical ripening in women with a prior cesarean delivery and an unfavorable cervix was done using various methods. Fifteen hospitals (21.4%) did not use cervical ripening in case of an unfavorable cervix but planned an elective repeat cesarean section (ERCS). Of the 55 hospitals that applied cervical ripening, 40 hospitals (72.7%) used mechanical methods; 2 hospitals used a hygroscopic cervical dilator (Dilapan-S), and 38 hospitals used a Foley catheter, including three hospitals using it in combination with oxytocin. Two of the 55 (3.6%) hospitals used a Foley catheter in combination with prostaglandins (oral E1 tablet 50 mcg *n* = 1, cervical prostaglandin E2 gel 0.5 mg *n* = 1), and two other hospitals (3.6%) used membrane sweeping or oxytocin only. Eleven hospitals (20%) used prostaglandin analogues. Specifications are shown in Table 4 including the frequency of administration and maximum daily doses, specified for the preferred method of cervical ripening. Seventeen hospitals (30%) used more than one method of cervical ripening. 

In 49% of the hospitals, the method of cervical ripening has changed over the past five years. The reason most frequently mentioned were “evidence from the literature concerning an elevated risk of uterine rupture with the use of prostaglandins” (*n* = 21) and “the reintroduction of the Foley catheter” (*n* = 5). 

According to the hospitals that applied cervical ripening in women with a prior cesarean delivery (*n* = 55), their motivations for the method of choice were “reduced hyperstimulation risk” (*n* = 12), “easy in use” (*n* = 6), “more deliveries in 24 hours” (*n* = 2), and “less fetal distress” (*n* = 1). Sixteen hospitals mentioned that their method was preferred “for different reasons”, and 13 gave a combination of reasons, 10 including “reduced hyperstimulation risk.” Five hospitals gave no motivation for the use of their preferred method. 

For the assessment of cervical ripeness and the consequent decision of cervical ripening and induction, 35 of the 55 hospitals (64%) used a cervical scoring system such as the Bishop score. Of the 35 hospitals that used the Bishop score, induction after cervical ripening by amniotomy and oxytocin augmentation was performed at a certain Bishop score (Figure 1). Out of the 20 hospitals that did not use a cervical scoring system, the decision to perform amniotomy was based on the ease in which the membranes could be reached in five hospitals. Six hospitals reported that a minimum of 2 cm dilatation was required to perform amniotomy. 

Continuous fetal monitoring was applied in 16 hospitals of which 10 used the Foley catheter and four used prostaglandin E2 gel. Amniotomy and subsequent oxytocin augmentation after proven cervical ripeness were performed at any moment of the day in 21 hospitals (38%), the morning after in nine hospitals (16%), and depended on the indication of induction in eight hospitals (15%). 

The policy after insufficient result of cervical ripening after one or two days is shown in Table 5. 

### 3.3. Comparison: Cervical Ripening in Women with and without a Prior Cesarean Delivery

When comparing cervical ripening in women with and without a prior cesarean delivery, fetal monitoring using CTG was conducted more frequently in women with a prior cesarean delivery. Women with a prior cesarean delivery were monitored continuously in 16 hospitals compared to two hospitals for women without a prior cesarean delivery. The timing of amniotomy and subsequent oxytocin augmentation hardly differed between women without or with a prior cesarean delivery. However, the timing of amniotomy and oxytocin augmentation in women with a prior cesarean delivery seemed less influenced by the indication of induction (15 versus 27%) or the presence of contractions (4 versus 11%). Two hospitals did not perform amniotomy after a specific time (16.00 hrs and 24.00 hrs) if the parturient was not in labor. 

The use of a cervical scoring system was nearly equal in women with (69%) and without (64%) a prior cesarean delivery. 

### 3.4. Comparison with Survey Conducted in 2006

Similar to the 2006 survey (response rate 94%, *n* = 77/82), there was a great variety in methods of cervical ripening in 2010. In women without a prior cesarean delivery, all hospitals in 2010 used prostaglandins as preferred method for cervical ripening, as was the case in 2006. 

Concerning women with a prior cesarean delivery, 72.7% (40/55) of the hospitals that allowed induction of labor used mechanical methods for cervical ripening in 2010 compared to 49.1% (28/57) in 2006 (*P* < 0.05). The Foley catheter specifically was used two and half times more often in 2010 (69.1% versus 25%, *P* < 0.01). The use of prostaglandins in cervical ripening in women with a prior cesarean decreased from 40% in 2006 to 20% in 2010 (*P* < 0.05). 

There was a nonsignificant decrease in the percentage of hospitals that did not apply cervical ripening and thus performed an ERCS in 2010 (21% versus 26%). 

Of the six hospitals that did not apply cervical ripening in 2006 and returned both the 2006 and 2010 surveys, four switched to Foley catheter use, one to dilapan and one to prostaglandin E2 gel in 2010. Of the twelve hospitals that did not apply cervical ripening in 2010 and returned both the 2006 and 2010 surveys, two hospitals used dilapan, one used intracervical prostaglandin E2 gel, and one used the foley catheter for cervical ripening in 2006. Twenty out of 21 hospitals that participated in the PROBAAT study in 2010 returned the survey, revealing that 70% (14/20) used the Foley catheter, 15% (3/20) used prostaglandins, and 15% (3/20) did not offer induction of labor in women with a prior cesarean delivery.

Although all respondents in 2006 felt that their method of cervical ripening was easiest in use and led to more deliveries in 24 hours, the respondents in 2010 mostly mentioned “easy in use” and “less hyperstimulation” in response to the question why their preferred method of cervical ripening was superior. This survey showed an increase in use of the Bishop score from 39% in 2006 to 69% in 2010 (*P* < 0.01).

## 4. Discussion

With a response rate of 78%, this survey gives a representative view of the current methods of cervical ripening in the Netherlands, showing a great variety in methods of cervical ripening. 

The use of intracervical prostaglandin gel has declined over the years, whereas the use of vaginal misoprostol has increased. It is striking that the Foley catheter is never used as the preferred method of cervical ripening, neither in 2006 nor in 2010. As the results of the PROBAAT trial 15 were not yet known, it is possible that hospitals were waiting for the results of this trial before changing their preferred method of cervical ripening. 

The decrease of ERCS and the increase in use of the Foley catheter in women with a prior cesarean delivery are most likely due to the decreased popularity of prostaglandins and the (re)introduction of the Foley catheter through recent randomized controlled trials including the PROBAAT trial [15, 19]. Furthermore, the results of the studies of Kwee et al. and Lydon-Rochelle et al., in which an increased risk of uterine rupture was found when prostaglandins were used in this group, may have discouraged the use of prostaglandins in these women [7, 9].

The nationwide variety in methods of cervical ripening is not surprising, especially when we bear in mind the lack of evidence and recommendations in the Dutch guideline on induction of labor in which different methods are mentioned to have similar effectiveness and safety profiles [4]. This may also be the basis for the use of more than one method for cervical ripening in 25 hospitals (36%), probably showing that tradition, culture, experience, and personal preference of gynecologists are of influence on methods used.

It seems that hospitals are increasingly concerned with safety of induction instead of the speed of delivery. However, standardisation of medical procedures and prescriptions reduces medication errors [20–22]. Therefore, a clearly defined, written local protocol on induction of labor including evidence-based methods of induction which are effective and safe would be advisable. 

The Dutch guideline on induction recommends the use of the Bishop score for cervical assessment, but also points out that it remains a subjective method [4]. In both surveys there is a great variation in the (Bishop) score at which the cervix is deemed favorable for amniotomy and subsequent oxytocin augmentation. 

All hospitals applied fetal monitoring at set times after starting cervical ripening, although length and frequency differed. Again, the lack of evidence and uniformity in the literature and in guidelines is reflected in the wide variety of clinical practice. While the guideline does not mention timing or frequency of fetal monitoring, all hospitals seem to have a local protocol to which they adhere.

Although amniotomy and subsequent oxytocin augmentation were performed at any moment of the day in half of the hospitals, a substantial part of hospitals waited until the next day to continue induction. Sometimes, it was also dependent on the indication for induction or the presence of contractions. Whether or not the attention in the media for the publication by de Graaf et al. on increased adverse perinatal outcome of hospital delivery at night influenced this decision remains unclear [23]. Furthermore, the Dutch guideline advocates an interval of 6 to 12 hours after cervical ripening using PGE2 analogues before performing amniotomy because of the risk of potentiation. These phenomena may be the basis for 29 hospitals (41%) to wait until the next day to continue induction and that two clinics discontinued after a certain hour unless strictly necessary. 

Although 28 hospitals answered positively to the question whether or not an unfavorable cervix was a reason not to induce labor in women with a prior cesarean delivery, only 15 hospitals declared not to apply cervical ripening in women with a prior cesarean delivery. Possibly the question was not written clearly or misread.

Limitations of this study are that this survey was sent to only one gynecologist per hospital, which may introduce bias. Results may be affected by the subjective perception and practice of that particular gynecologist. However, the responding gynecologist was asked to answer on behalf of the staff and based on protocols or policies.

It remains unclear what the maximum period of induction was for the different hospitals; that is, which definition of failed induction they used. At the time of the survey, several hospitals (28.5% of the respondents) participated in the PROBAAT trial, in which failed induction was considered after 4 days. It is likely that the participating hospitals of the PROBAAT trial used this definition [15].

The incidence of uterine rupture in 2002 in the Netherlands was 0.8% in women with a prior cesarean delivery undergoing a trial of labor without contraction-stimulating drugs and 1.47% in all women [7]. Another Dutch study reported an estimated risk of uterine rupture to be 0.64% in these women undergoing a trial of labor between 2004 and 2006 [24]. Although several studies suggest an increased risk of uterine rupture in the use of PGE2 analogues, a review in 2006 did not convey a negative advice due to lack of evidence [9, 25–27]. The Dutch guideline does not discourage their use but merely suggests that the elevated risk of uterine rupture when using contraction-stimulating drugs should be weighed and discussed with the women [8]. It is remarkable that in the case of cervical ripening of women with a prior cesarean delivery, policies concerning induction of labor differ so greatly between hospitals. 

## 5. International Comparison of Induction of Labor in Women without or with a Prior Cesarean Delivery

We were unable to identify other surveys of practice concerning methods of cervical ripening in women without a prior cesarean delivery.

Comparison of the results of our study with surveys from the UK, Australia New Zealand, and Canada shows that the use of prostaglandins for cervical ripening in women with a prior cesarean delivery is by far the lowest in the Netherlands (20%) [16–18]. Also, cervical ripening using mechanical methods in this group is much more popular in the Netherlands (73%) compared to England (3%) (Table 6). 

 However, it should be noted that the data span 2003 to 2011 making this comparison difficult since many changes in practice concerning prostaglandin use in women with a prior cesarean delivery have been made over the last decade.

We recommend repeating this survey (inter)nationally to assess whether or not results of the PROBAAT trial have influenced policy concerning cervical ripening.

## 6. Conclusions

There is a large diversity in methods of cervical ripening in the Netherlands. In women without a prior cesarean delivery prostaglandins are most frequently used, which is in line with other countries. In women with a prior cesarean delivery, the Foley catheter is most often used, which is in contrast to other high-income countries where prostaglandins are mainly used. Although the Foley catheter has become more popular in cervical ripening in women with a prior cesarean delivery, the overall policy in these women is still diverse, and prospective comparisons of different induction methods in these women are lacking. We conclude that a study concerning safety and effectiveness of the Foley catheter for cervical ripening in women with a prior cesarean delivery is recommended.

## Figures and Tables

**Table 1:** Overview of survey respondents according to type of hospital in The Netherlands in 2010.

Type of hospital	Number of returned surveys
University hospital	7/8
Teaching hospital	31/37
District hospital	31/47

Total number of respondents is 70; one survey was returned anonymously.

**Table 2:** Methods of cervical ripening in women without a prior cesarean delivery in The Netherlands in 2010.

	Intracervical prostaglandin E2 gel (Prepidil) 0.5 mg	Intravaginal prostaglandin E2 gel (Prostin) 1 mg	Intravaginal prostaglandin E2 gel (Prostin) 2 mg	Intravaginal prostaglandin E2 gel (Prostin) 1 + 2 mg	Slow release vaginal insert prostaglandin E2 (Propess)	Intravaginal prostaglandin E2 tablet (Prostin) 3 mg	Intravaginal prostaglandin E1 misoprostol tablet (Cytotec)	Oral prostaglandin E1 misoprostol tablet (Cytotec)	Foley catheter	Foley catheter + prostaglandins (E1 or E2)
*Administration f* *re* *qu* *en* cy*										
Once	2	—	2	—	14	—	—	—	—	2
Every 2 h	0	—	—	—	—	—	—	1	—	—
Every 4 h	2	—	4	7	—	—	9	—	—	—
Every 6 h	2	4	5	11	—	2	—	—	—	1
Every 12 h	—	—	1	—	—	—	—	—	—	—

*Maximum daily d* *os* es*										
Once	1	—	1	—	14	—	—	—	—	1
Twice	4	2	10	12	—	—	—	—	—	—
3 times	—	2	2	5	—	2	8	—	—	2
4 times	1	—	—	1	—	—	1	—	—	—
6 times	—	—	—	—	—	—	—	1	—	—

Total *n* = 70	6	4	13	18	14	2	9	1	—	3

Second choice *n* = 26	1	1	—	—	2	—	2	—	19	1

*Concerns the preferred method of cervical ripening.

**Table 3:** Policy after insufficient result of cervical ripening after one and two days in women without a prior cesarean delivery in The Netherlands in 2010.

	Continue using same method without day(s) of rest	Day of rest after 1 day, then continue using same method	Day of rest after 2 days, then continue using same method	Day of rest after 2 days, then continue using different method	Foley catheter on day 3	Foley catheter on day 4	Other
Intracervical PG* E2 gel (Prepidil) 0.5 mg	3	1	1	1	—	1	—
Intravaginal PG* E2 gel (Prostin) 1 mg	1	—	3	—	—	—	—
Intravaginal PG* E2 gel (Prostin) 2 mg	2	—	6	5	—	3	—
Intravaginal PG* E2 gel (Prostin) 1 + 2 mg	5	—	13	—	—	—	—
Slow release vaginal insert PG* E2 (Propess)	4	2	7	—	2	—	1
Intravaginal PG* E2 tablet (Prostin) 3 mg	—	—	1	—	—	—	1
Intravaginal PG* E1 misoprostol tablet (Cytotec)	4	—	4	1	—	1	—
Oral PG* E1 misoprostol tablet (Cytotec)	—	—	—	1	—	—	—
Foley catheter combined with PG*(E1 or E2)	3	—	—	—	—	—	—

Total *n* = 70	22	3	35	8	2	5	2

*PG: prostaglandin.

**Table 4:** Methods of cervical ripening in women with a prior cesarean delivery in The Netherlands in 2010.

	Intravaginal prostaglandin E2 gel (Prostin) 1 mg	Intravaginal prostaglandin E2 gel (Prostin) 1 + 2 mg	Slow release vaginal insert prostaglandin E2 (Propess)	Intravaginal prostaglandin E1 misoprostol tablet (Cytotec)	Foley catheter	Foley catheter + prostaglandines (E1 or E2)	Foley catheter + oxytocin	Hygroscopic cervical dilator (Dilapan-S)	Elective repeat cesarean section	Other: sweep (1), oxytocin (1)
*Frequency of * administration*										
Once	1	—	2	—	35	2	3	1	n/a	n/a
Every 4 h	1	2	—	1	—	—	—	—	n/a	n/a
Every 6 h	3	1	—	—	—	—	—	—	n/a	n/a
Every 12 h	—	—	—	—	—	—	—	1	n/a	n/a

*Maximum daily* dosis*										
Once	1	—	2	—	35	2	3	1	n/a	n/a
Twice	2	1	—	—	—	—	—	1	n/a	n/a
Three times	2	2	—	1	—	—	—	—	n/a	n/a

Total *n* = 70	5	3	2	1	35	2	3	2	15	2

Second choice *n* = 23	3	3	2	—	6	—	—	6	3	—

*Concerns the preferred method of cervical ripening. n/a: not applicable.

**Table 5:** Policy after insufficient success of cervical ripening after one and two days in women with a prior cesarean delivery in The Netherlands in 2010.

	Not applicable	Continue using same method without day(s) of rest	Day of rest after 1 day, then continue using same method	Day of rest after 2 days, then continue using same method	Day of rest after 2 days, then continue using different method	Cesarean section on day 2	Cesarean section on day 3	Unclear or unknown	Total
None, elective repeat cesarean section	15	—	—	—	—	—	—	—	15
Intravaginal PG* E2 gel (Prostin) 1 mg	—	1	—	2	1	—	1	—	5
Slow release vaginal insert PG* E2 (Propess)	—	1	1	—	—	—	—	—	2
Intravaginal PG* E2 gel (Prostin) 1 + 2 mg	—	—	—	2	—	1	—	—	3
Intravaginal PG* E1 misoprostol tablet (Cytotec)	—	—	—	1	—	—	—	—	1
Foley catheter	—	8	—	9	1	7	8	2	35
Foley catheter combined with PG*	—	1	—	—	—	—	—	1	2
Foley catheter with oxytocin	—	2	—	—	—	1	—	—	3
Hygroscopic cervical dilator (Dilapan-S)	—	—	—	—	—	—	2	—	2
Other (sweeping or oxytocin)	—	1	—	—	—	1	—	—	2

Total	15	14	1	14	2	10	11	3	70

*PG: prostaglandin.

**Table 6:** Comparison of international surveys of current practice of induction of labour in women with a prior cesarean birth.

	Response rate	Repeat CS (i.e., no induction of labour)	Use of prostaglandins	Use of mechanical methods	Use of ARM	Willingness to use oxytocin
England 2011* [16]	67% (322/480)	7% (22/322)	76% (229/300)	3% (9/300)	21% (62/300)	unknown
Australia, NZ 2003** [17]	67% (1091/1641)	32% (349/1091)	33% (360/1091)	unknown	unknown	73% (796/1091)
Canada 2003*** [18]	50% (750/1497)	9% (54/601)	25% (150/601)	unknown	unknown	unknown
The Netherlands 2010	78% (70/92)	21% (15/70)	20% (11/55)	73% (40/55)	4% (2/55)	unknown

NZ: New Zealand; CS: cesarean section; ARM: artificial rupture of membranes.

*Among 480 NHS obstetric consultants.

**Among fellows and members of the Royal Australian and New Zealand College of Obstetricians and Gynecologists.

***Among all obstetricians registered with the Canadian Medical Directory.
